# Supplementary figures and images for: An immunohistochemical prostate cell identification key indicates that aging shifts procollagen 1A1 production from myofibroblasts to fibroblasts in dogs prone to prostate-related urinary dysfunction
Source: PLoS One. 2020 Jul 29;15(7):e0232564. doi: 10.1371/journal.pone.0232564 (PMC7390344; doi:10.1371/journal.pone.0232564)

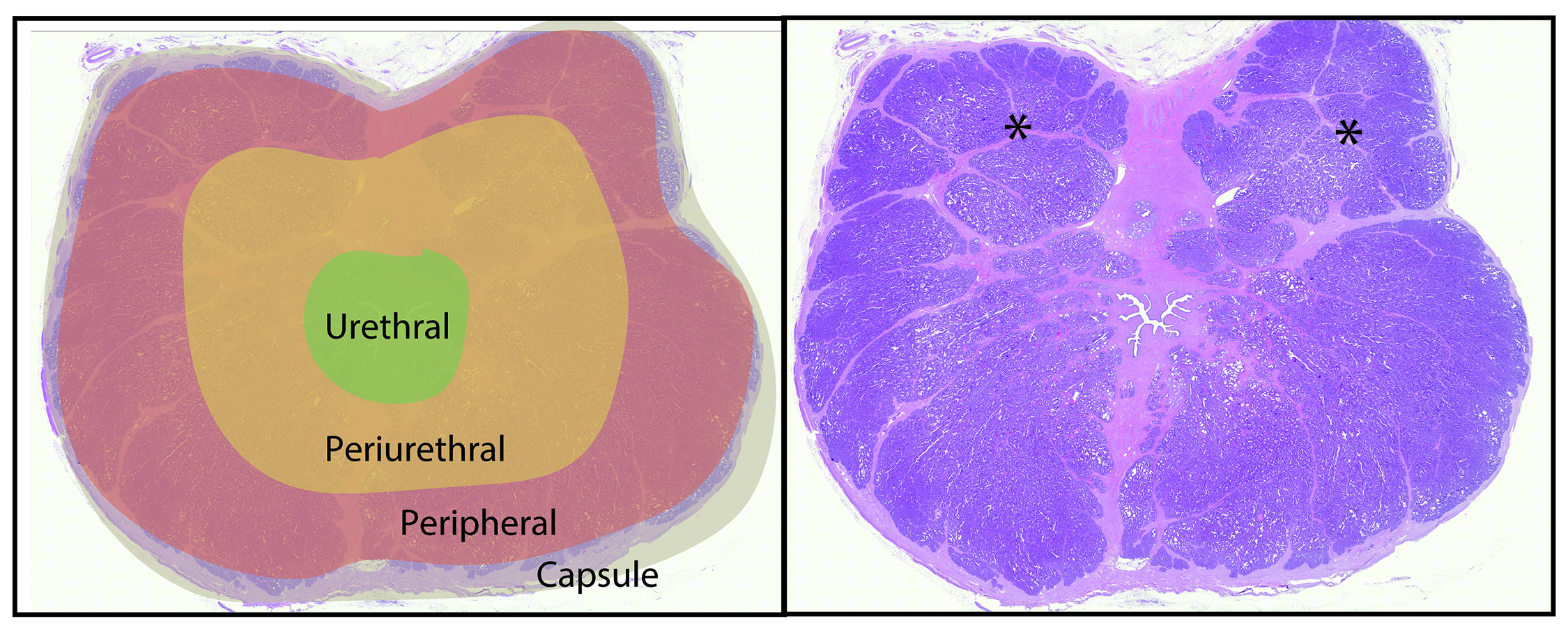

Supplement: S1 Fig — Shown is a tile scan of a hematoxylin and eosin stained prostate of a young intact male dog overlaid with regions (left) and shown in the same scale (right). The urethral region is defined by the urethral epithelium and its associated stroma (circumferential orientation of stromal fibers). The capsule is the stroma including large vessels and large bundles of muscle comprising the outer boundary of the prostate and is void of epithelial cells. The periurethral and peripheral regions do not have clear anatomical boundaries and are defined by dividing the tissue between the capsule and urethra into two rings. This particular prostate is a rare example of a (*) thin band of stroma running circumferentially and separating the two zones in the dorsal portion of the gland). High resolution images shown here and others from biological replicates are available through the GUDMAP database at https://doi.org/10.25548/16-WMM4. (TIF) [file pone.0232564.s001.tif]

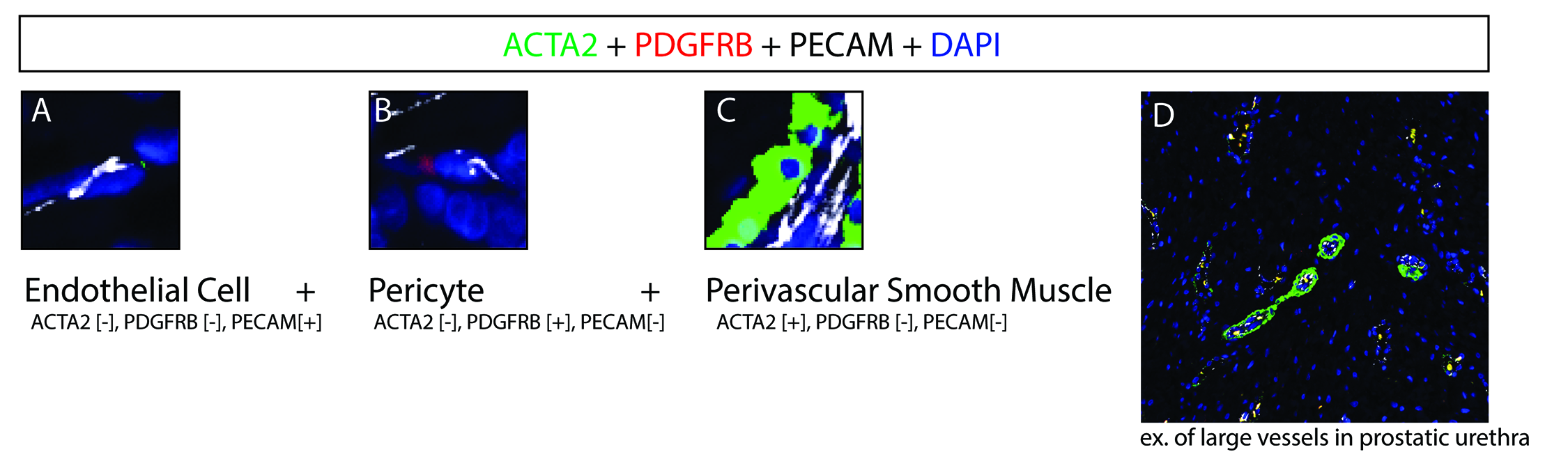

Supplement: S2 Fig — Multiplex immunostaining with antibodies against smooth muscle actin (ACTA2), Platelet derived growth factor receptor beta (PDGFRB) and Platelet and endothelial cell adhesion molecule (PECAM) was performed on complete transverse sections of the young (≤3 years old) intact male canines. Nuclei were stained with DAPI. (A-C) Endothelial cells, pericytes, and perivascular smooth muscle cells were identified based on combinatorial staining. Endothelial cells, pericytes, and perivascular smooth muscle cells were visualized within each region (capsule depicted in D). Images are representative of 9 young intact male dog prostates. High resolution images shown here and others from biological replicates are available through the GUDMAP database at https://doi.org/10.25548/16-WMM4. (TIF) [file pone.0232564.s002.tif]

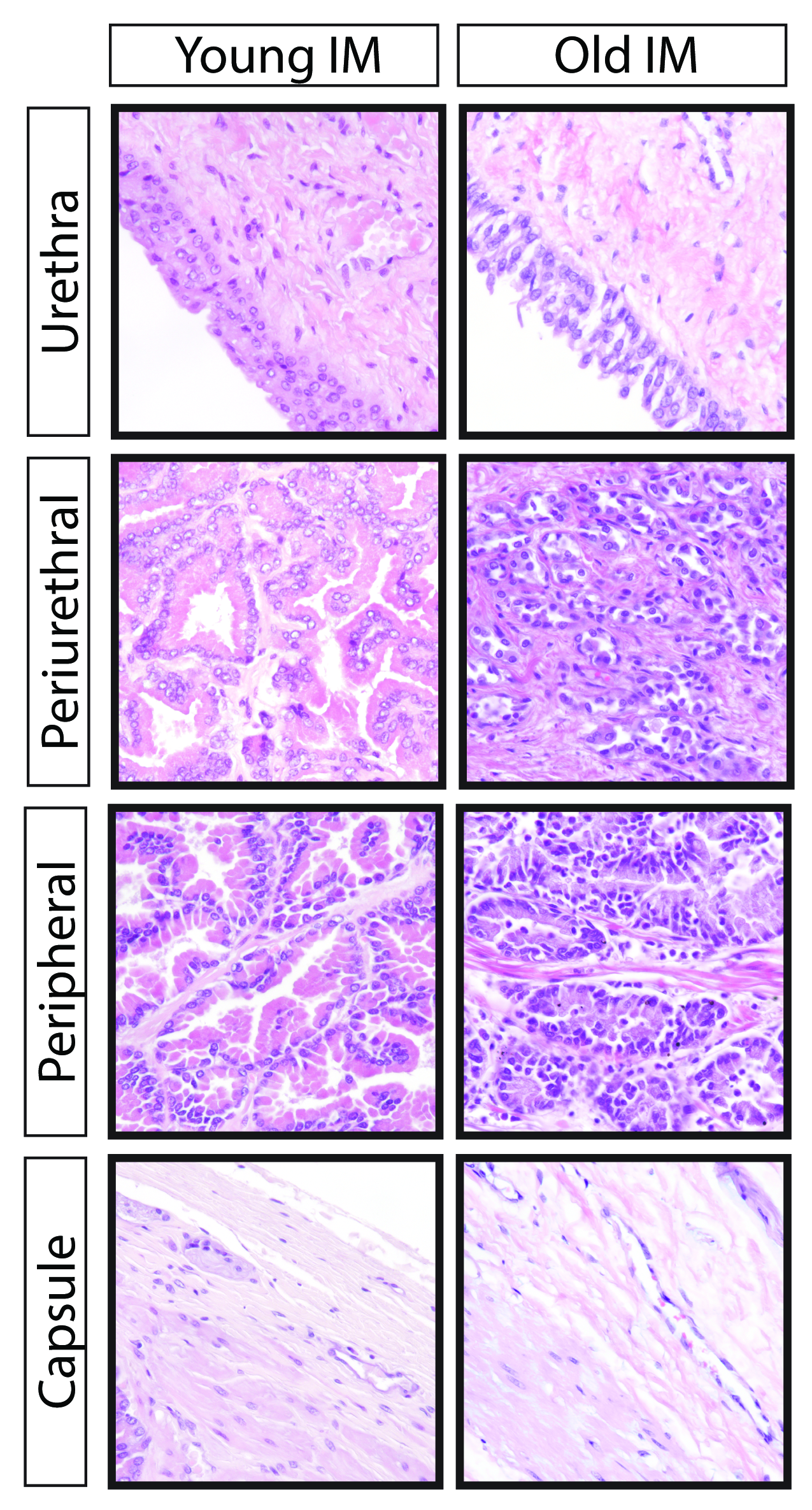

Supplement: S3 Fig — Sections from young and old intact male dogs were stained with hematoxylin and eosin. Results are representative of 9 young and 4 old intact male dog prostates. High resolution images shown here and others from biological replicates are available through the GUDMAP database at https://doi.org/10.25548/16-WMM4. (TIF) [file pone.0232564.s003.tif]

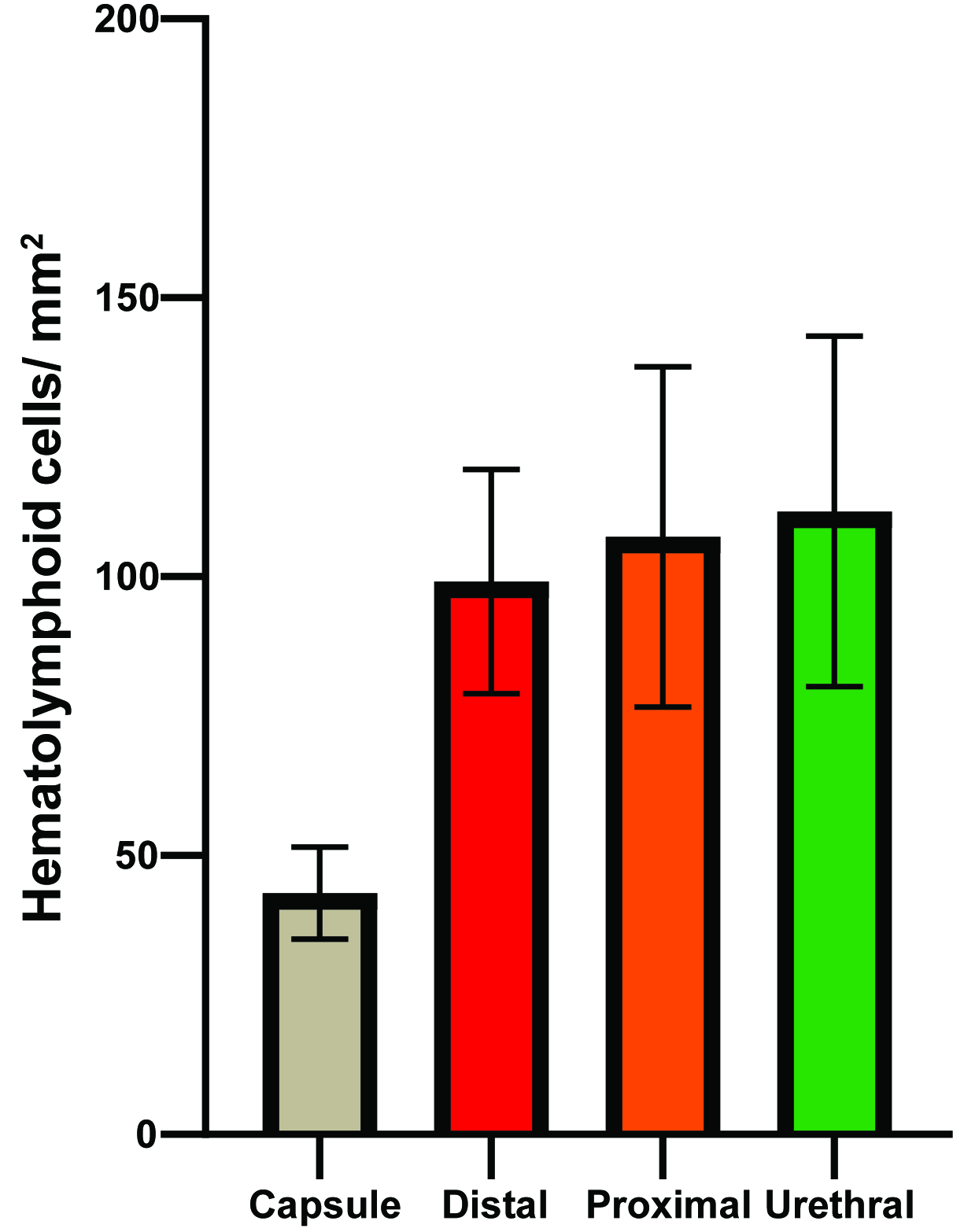

Supplement: S4 Fig — Sections from young intact male dogs were immunostained with an antibody against protein tyrosine phosphatase, receptor type C (PTPRC). Nuclei were stained with DAPI. Hematolymphoid cells (PTPRC+) were visualized, and densities quantified within each region. Results are mean ± SE of 6–8 young intact male dog prostates, a one-way ANOVA was used to compare regions, no statistical differences were found between regions. High resolution images shown here and others from biological replicates are available through the GUDMAP database at https://doi.org/10.25548/16-WMM4. (TIF) [file pone.0232564.s004.tif]

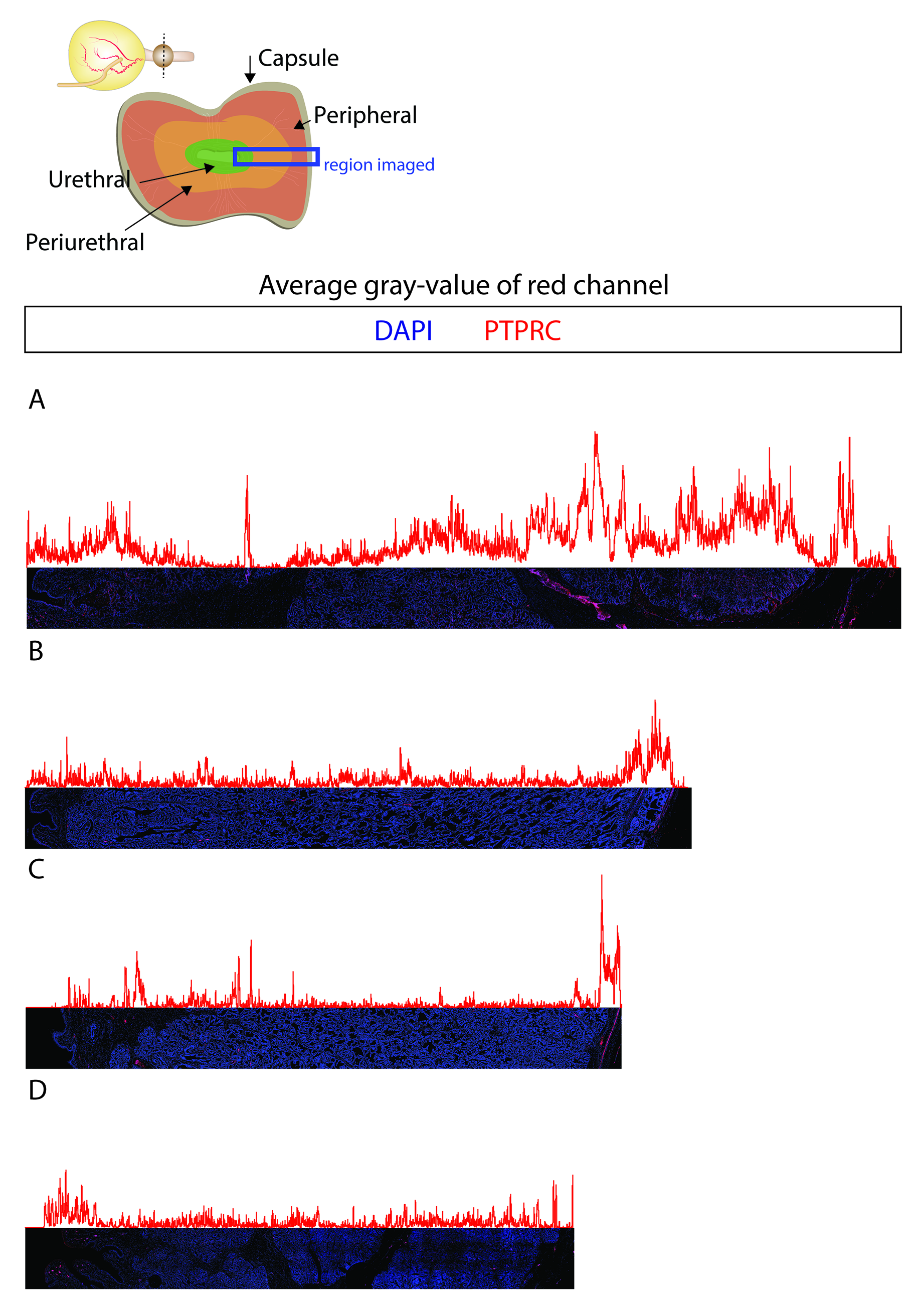

Supplement: S5 Fig — Four prostates from young intact males were tile scanned in three 20X field tall strip from the urethra to the capsule in the right or left lateral portion of the gland (A-D). We used image J- plot profile to determine the average gray value for each column of pixels, creating a plot of the urethral to capsule distribution of hematolymphoid cells in the prostate (A-D). Using this technique, we found that hematolymphoid cells were not distributed in a proximal to distal pattern but rather distributed fairly ubiquitously throughout the prostate. High resolution images shown here and others from biological replicates are available through the GUDMAP database at https://doi.org/10.25548/16-WMM4. (TIF) [file pone.0232564.s005.tif]
